# Unprimed, M1 and M2 Macrophages Differentially Interact with *Porphyromonas gingivalis*

**DOI:** 10.1371/journal.pone.0158629

**Published:** 2016-07-06

**Authors:** Roselind S. Lam, Neil M. O’Brien-Simpson, James A. Holden, Jason C. Lenzo, Shao B. Fong, Eric C. Reynolds

**Affiliations:** Oral Health Cooperative Research Centre, Melbourne Dental School, Bio21 Institute, The University of Melbourne, Melbourne, Australia; Medical University of South Carolina, UNITED STATES

## Abstract

*Porphyromonas gingivalis* is a keystone pathogen in the development of chronic periodontitis. Tissue macrophages are amongst the first immune cells to respond to bacteria and depending on the cytokine profile at the infection site, macrophages are primed to react to infection in different ways. Priming of naive macrophages with IFN-γ produces a classical pro-inflammatory, antibacterial M1 macrophage after TLR ligation, whereas priming with IL-4 induces an anti-inflammatory tissue-repair M2 phenotype. Previous work has shown that M1 are preferentially generated in gingival tissue following infection with *P*. *gingivalis*. However, few studies have investigated the interactions of macrophage subsets with *P*. *gingivalis* cells. The aim of this study was to determine the ability of naive, M1 and M2 macrophages to phagocytose *P*. *gingivalis* and investigate how this interaction affects both the bacterial cell and the macrophage. M1 and M2 macrophages were both found to have enhanced phagocytic capacity compared with that of naive macrophages, however only the naive and M1 macrophages were able to produce a respiratory burst in order to clear the bacteria from the phagosome. *P*. *gingivalis* was found to persist in naive and M2, but not M1 macrophages for 24 hours. Phagocytosis of *P*. *gingivalis* also induced high levels of TNF-α, IL-12 and iNOS in M1 macrophages, but not in naive or M2 macrophages. Furthermore, infection of macrophages with *P*. *gingivalis* at high bacteria to macrophage ratios, while inducing an inflammatory response, was also found to be deleterious to macrophage longevity, with high levels of apoptotic cell death found in macrophages after infection. The activation of M1 macrophages observed in this study may contribute to the initiation and maintenance of a pro-inflammatory state during chronic periodontitis.

## Introduction

Chronic periodontitis is an inflammatory disease of the supporting tissues of the teeth characterised by accumulation of immune cells in gingival tissues leading to bone resorption, eventuating in tooth loss [1]. It affects around 30α of adults and has been linked to a higher risk of certain systemic diseases such as cardiovascular, diabetes, respiratory infections, pancreatic cancer, spontaneous pre-term birth and pre-term low birth weight [2–5]. *Porphyromonas gingivalis* is one of the bacterial biofilm species isolated from subgingival plaque most strongly associated with clinical indicators of periodontitis, including increased pocket depth and bleeding on probing [6, 7]. *P*. *gingivalis* has been shown to be a keystone pathogen that causes disease following host flora dysbiosis [8]. The level of *P*. *gingivalis* in subgingival plaque above threshold levels (>10% of total bacterial load) has been shown to predict imminent disease progression in periodontitis patients [9]. The extracellular Arg- and Lys-specific cysteine proteinases, outer membrane vesicles and variation in lipopolysaccharides (LPS) structure have been reported to be some of *P*. *gingivalis* major virulence factors that can induce variable immune responses, leading to dysregulated immunity [10–12].

The monocyte-macrophage system has a crucial role in innate immunity and also in the initiation of the adaptive immune response [13]. Macrophages, as long-lived tissue resident cells, are one of the first immune cells to encounter antigens from invading organisms [14]. One of the commonly observed characteristics in chronic periodontitis is the presence of macrophages in the localised diseased tissues [13, 15, 16]. Elevated numbers of macrophages and pro-inflammatory cytokines interleukin (IL)-1, tumour necrosis factor alpha (TNF-α), macrophage inflammatory protein 1-alpha (MIP-1α) and IL-8 secreted by macrophages have been detected in gingival tissue biopsies from chronic periodontitis patients [15, 17].

As macrophages are recruited to the site of infection, they are exposed to a variety of stimuli; both host derived cytokines and chemokines as well as ligands derived from pathogens. Exposure of naive monocytes to host cytokines, such as interferon gamma (IFN-γ) or IL-4, prior to toll-like receptor (TLR) ligation gives rise to a variety of different macrophage phenotypes. Many macrophage subsets have now been described and there have been attempts to provide a comprehensive framework of nomenclature based on stimuli rather than cellular phenotype [18]. According to the proposed nomenclature, the experiments described here use M(-), M(LPS), M(IFN-γ), M(IFN-γ + LPS), M(IL-4) and M(IL-4 + LPS). However, for consistency with our previous studies we will use naive, pre-M1, M1, pre-M2 and M2 for this investigation. Although TLR stimulation is not generally necessary to generate a M2 macrophage phenotype, the inclusion of it in these experiments completes all possible permutations of cytokine and TLR stimulant additions. Our previous work has shown that the addition of PAM3CSK4 generates a M1 macrophage phenotype as effectively as the addition of *Escherichia coli* LPS [19]; however this flexibility in TLR stimuli is not reflected in the current nomenclature proposals and is further complicated by the addition of mixed TLR ligands such as those found on bacterial whole cells.

Priming naive macrophages with IFN-γ prior to TLR ligation generates a M1 macrophage phenotype. M1 macrophages express CD86/CD80 proteins required for T-cell priming and produce IL-1, IL-12, IL-23 and TNF-γ, bactericidal reactive oxygen intermediates and catabolise arginine into nitric oxide (NO). M2 macrophages are induced by IL-4 or IL-13 cytokines; however do not necessarily require TLR stimulation. M2 macrophages shift the metabolism of arginine away from NO and towards the collagen precursor _L_-ornithine (with urea as a by-product) with anti-humoral (anti-inflammation) antibody production–contributing towards a tissue repair phenotype. IL-13 was initially thought to be functionally redundant with IL-4, where both cytokines exert anti-inflammatory effects on other cells [20]. However, recent experiments involving IL-13 knockout mice and the use of IL-13 neutralising antibodies have demonstrated non-redundant roles for IL-13 particularly in its effect on the monocyte-macrophage system [20–22].

We have previously shown that M1 macrophages are the dominant gingival infiltrating macrophage in response to *P*. *gingivalis* oral infection in the mouse periodontitis model [23]. Purified *P*. *gingivalis* LPS was able to generate a M1 macrophage phenotype, inducing CD86 expression, nitric oxide synthesis and inflammatory cytokine production, although this activation was not as strong as with *E*. *coli* LPS. *P*. *gingivalis* LPS did not strongly induce markers of M2 macrophage production beyond those induced by IL-4 itself. While macrophages appear to accumulate in diseased gingival tissues of patients with chronic periodontitis and the diseased tissue contains a variety of cytokines that would influence the activated macrophage phenotype, a direct link to periodontal pathogens stimulating M1 and M2 in periodontitis has not been shown. Furthermore, little information beyond cytokine production is available regarding the ability of whole *P*. *gingivalis* cells to generate M1 and M2 macrophage phenotypes, nor has there been an investigation into how cytokine priming affects macrophage phagocytosis of *P*. *gingivalis*.

Priming of macrophages with IFN-γ or IL-4 prior to TLR ligation has considerable consequences for how macrophages detect, phagocytose and kill bacteria. Exposure of macrophage to IL-4 prior to *Neisseria meningitidis* demonstrated a significant reduction of the uptake of the bacteria [24], but also resulted in an increase in phagosomal acidity and cathepsin activity [25]. These features are essential for the efficient proteolytic cleavage of proteins, both host and foreign, but does not necessarily favour the destruction of intact bacteria [25]. Furthermore, M2 macrophages upregulate the expression of scavenger receptors such as CD206, CD163 and Macrophage Receptor with Collagen Structure (MARCO), which are all involved in the phagocytosis and clearance of cellular debris [24, 26]. Alternatively, priming with IFN-γ has been shown to increase the phagocytic capacity of the cells whilst delaying fusion of the phagosome with the lysosome whilst increasing the production of anti-microbial compounds, creating a phagosomal environment toxic to the bacteria yet conducive for the proteolytic generation of peptides appropriate for MHC presentation [27]. Treatment of macrophages with IL-4 or IFN-γ also has polar effects on the expression of Fc receptors, with IL-4 treatment increasing the balance of FcγRIIb, which signals through an inhibitory ITIM motif, whereas IFN-γ shifts the balance towards activatory ITAM signalling FcγRI and FcγRIII molecules. All the above mechanisms have significant implications for not just the phagocytosis of pathogens but the downstream processing of molecules derived from those pathogens and the subsequent inflammation induced.

Hence, understanding how the different macrophage phenotypes phagocytose *P*. *gingivalis* and subsequently interact with the bacterium should provide valuable insight into their potential role in *P*. *gingivalis*-related disease. In the present study therefore, we examined the effect of priming macrophages with IFN-γ and IL-4 on their phagocytic ability, the survival of *P*. *gingivalis* within such activated macrophages and the inflammatory response induced.

## Results

### M1 and M2 macrophages phagocytose a higher number of *P*. *gingivalis* W50 than naive macrophages

To examine the effect of IFN-γ or IL-4 on the phagocytosis of *P*. *gingivalis*, RAW264 cells were incubated with or without IFN-γ or IL-4 overnight. The primed macrophages were incubated with pHrodo-Red labelled *P*. *gingivalis* for 15 min (early time point) and 60 min (late time point) before flow cytometry analysis. The pHrodo-Red (fluorochrome) is pH sensitive and will not fluoresce in extracellular or cytoplasmic pH, hence fluorescence will only be detected once the pHrodo-Red labelled *P*. *gingivalis* are phagocytosed and the phagosome is acidified. This gives specific signals that consistently outperform quench-based phagocytosis assays.

**Fig 1:**
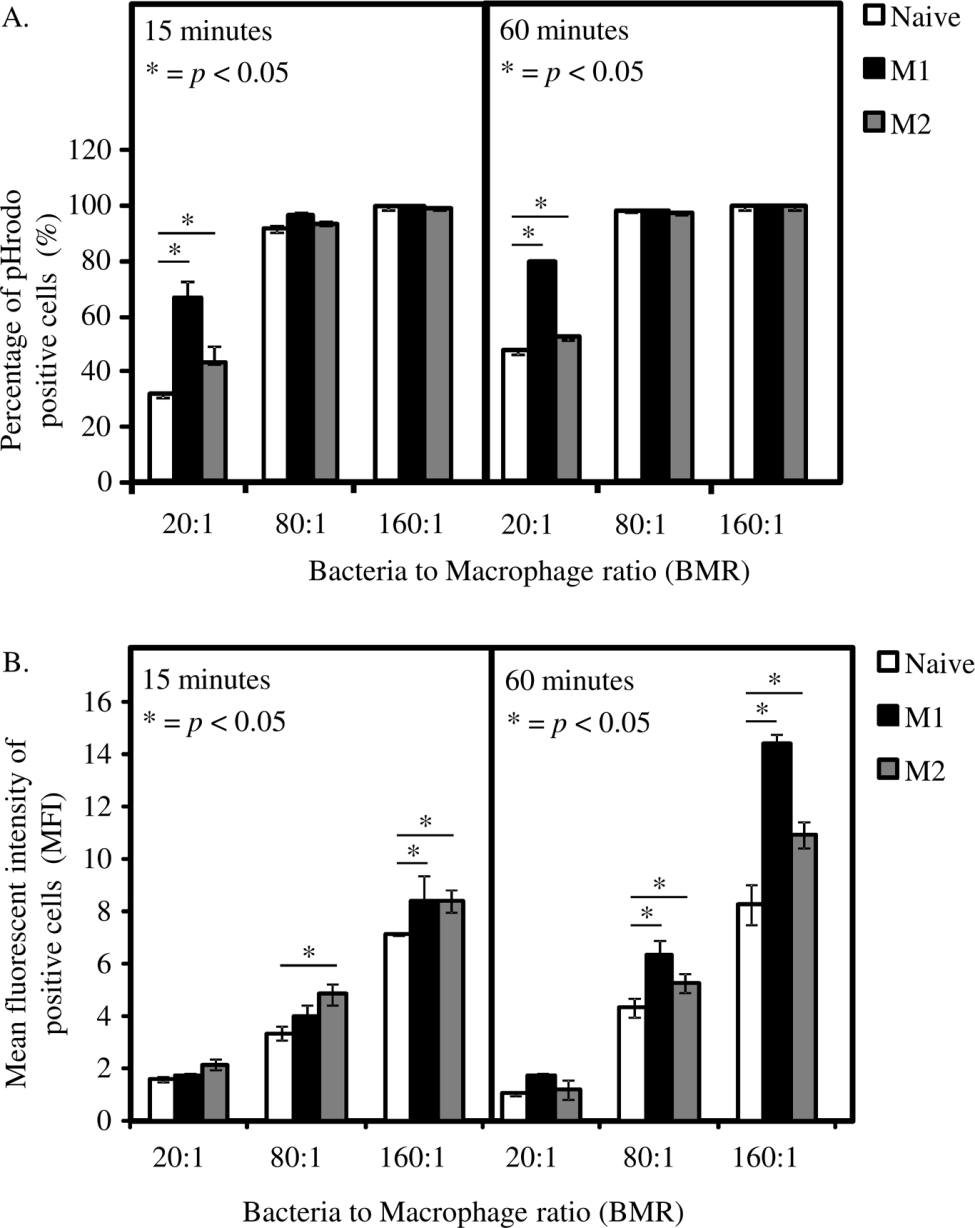
M1 and M2 macrophages phagocytose a higher number of *P*. *gingivalis* W50 than naive macrophages. In order to examine the effect of cytokines on phagocytosis, macrophages were incubated with either IFN-γ (M1) or IL-4 (M2) and incubated with pHrodo-Red labelled *P*. *gingivalis* W50 (bacteria to macrophage ratio 20:1, 80:1 and 160:1) for 15 min or 60 min prior to flow cytometric analysis. Data are expressed as the percentage of pHrodo positive cells **(A)** and its corresponding Mean Fluorescent Intensity (MFI) **(B)**. Data are representative of independent experiments and triplicate biological samples are expressed as the mean ± standard deviation (n = 3) and were analysed using student's t-test. * indicates data that are significantly different (*p* < 0.05).

The percentage of phagocytosed *P*. *gingivalis* increases in proportion to the number of *P*. *gingivalis* to macrophage ratio (bacteria to macrophage ratio, BMR) with 100% saturation at BMR 80:1 across all phenotypes at both time points (**Fig 1A**). At a lower BMR (20:1), a significantly higher proportion of M1 macrophages had phagocytosed *P*. *gingivalis* compared with naive and M2 macrophages at both 15 and 60 min. Macrophages primed with IL-4 also phagocytosed significantly higher levels of bacteria compared with naive macrophages, although significantly lower amounts compared with M1 macrophages. At higher BMRs, the fluorescent signal had shifted 100% of the macrophage population into the pHrodo-positive gate, and Mean Fluorescent Intensity (MFI) was then be used to distinguish populations. Based on the MFI at the later time point, M1 macrophages phagocytosed significantly more bacteria per cell than naive macrophages, as did M2 macrophages, although to a lesser extent (**Fig 1B**).

**Fig 2:**
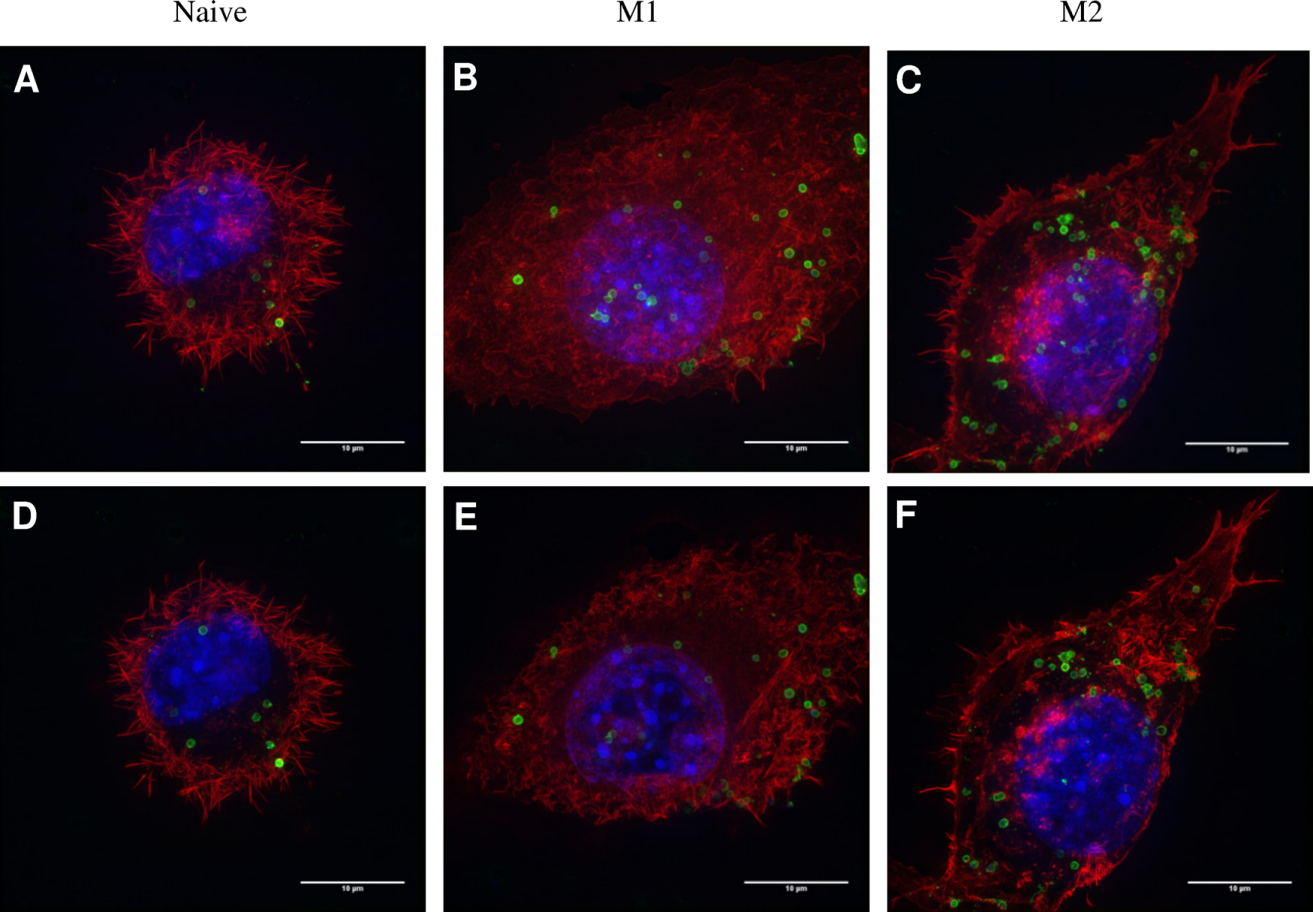
3D-SIM images of *P*. *gingivalis* W50 phagocytosed by naive, M1 and M2 macrophages. **(A,B,C)** Maximum intensity projection and **(D,E,F)** the corresponding slice view 3D-SIM images of *P*. *gingivalis* W50 phagocytosed by naive, M1 and M2 macrophages (60:1 BMR, 1 hour exposure time), respectively, on the Deltavision OMX Structured Illumination Microscope V4 Blaze (Applied Precision, WA, USA). *P*. *gingivalis* W50 were labelled with AF488 (green) while the nuclei and actin filaments of the macrophages were stained with DAPI (blue) and Phalloidin-TRITC (red) respectively. The slice images were obtained from a section with defined size (between 10–50 z-stack) to confirm phagocytosis of *P*. *gingivalis* W50 by naive, M1 and M2 macrophages.

As pHrodo is reliant on the acidification of the phagosome to generate fluorescence, super-resolution microscopy was used with AF488 labelled *P*. *gingivalis* to determine the cellular localisation of the bacterium. *P*. *gingivalis* W50-labelled with AF488 were incubated with naive, pre-M1 and pre-M2 macrophages and subjected to super-resolution imaging (**Fig 2**). Naive macrophages exhibited a rounded structure with strong, regular actin filaments, with some phagosomes containing fluorescent *P*. *gingivalis* observed. M1 macrophages exhibited a much more diffuse actin filament staining and “flattened” morphology with a proportionally larger cytoplasm, and a much higher amount of positive fluorescent bacteria than naive macrophages. M2 macrophages exhibit a spindle, fibroblast-like morphology with an enlarged cytoplasm and a high number of fluorescent bacteria. It is clear from the images that M1 and M2 macrophages both phagocytosed more *P*. *gingivalis* than naive macrophages, however, the M2 macrophages appeared to be associated with more bacteria than the M1 macrophages which was not consistent with the pHrodo flow cytometry data. The inclusion of Z-slices (**Fig 2D, 2E and 2F**) showed that the majority of the cell associated bacteria were internalised and not adhered to the surface of the macrophage. However, it should be noted that microscopy does not limit the localisation of the bacteria to a phagosome as with the pHrodo assay.

### M1 Macrophages are able to effectively phagocytose and kill intracellular bacteria

**Fig 3:**
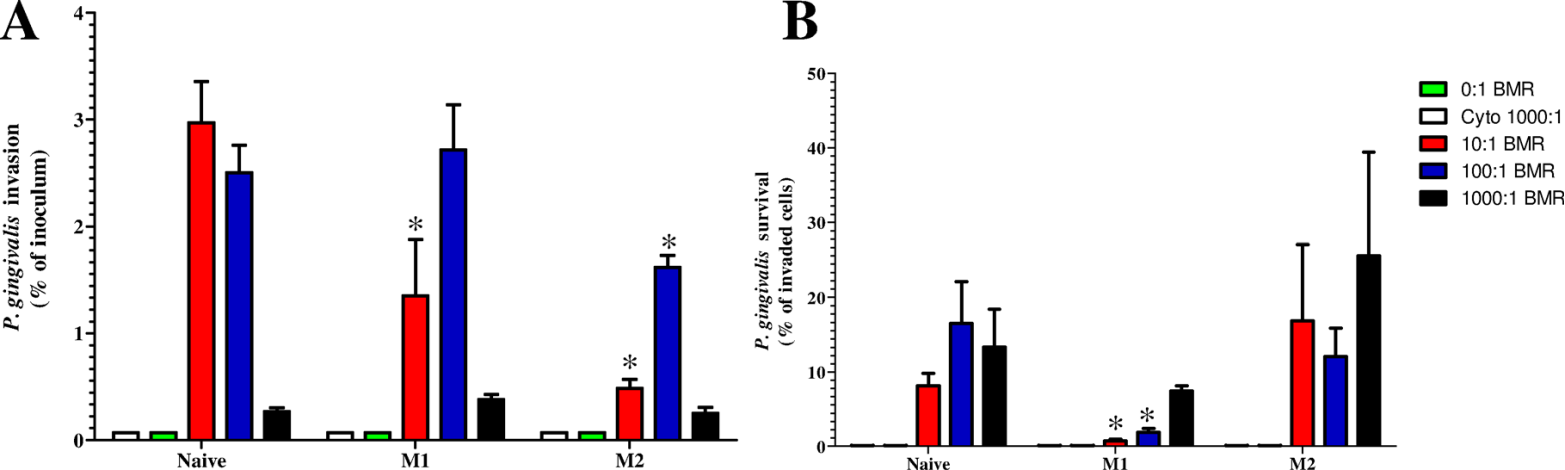
*P*. *gingivalis* is unable to survive M1 macrophage but persists within naive and M2 macrophages. Naive, M1 and M2 macrophages were incubated with *P*. *gingivalis* W50 (Pg) for 60 minutes at 37°C (Bacteria to cell ratio 10:1, 100:1 and 1000:1). After incubation, external bacteria were killed by incubation in gentamicin and metronidazole. The naive, M1 and M2 macrophages with internalised bacteria were then washed and lysed at 1- (invasion **Fig 3A**) and 24- hour (survival **Fig 3B**) post infection with bacteria. The *P*. *gingivalis* cell numbers recovered were then enumerated by culture. Cytochalasin B was added as negative control as it stabilises microtubules and inhibits actin polymerisation, a process required for phagocytosis. Invasion data are expressed as a percentage of the initial inoculum. Survival data are expressed as a percentage of the invaded bacteria. Data are representative of independent experiments and triplicate biological samples are expressed as the mean ± standard deviation (n = 3) and were analysed using a two-way ANOVA. * indicates significant (*p* < 0.05) differences between M1 or M2 macrophages and naive macrophages.

In order to determine the fate of phagocytosed *P*. *gingivalis* in M1 or M2 macrophages, both an invasion (1 hour) and survival assay (24 hours) were performed (**Fig 3**). Macrophages were incubated with live *P*. *gingivalis* for 60 minutes then extracellular bacteria killed by adding extracellular antibiotics to the media. Macrophages were lysed at 1 hour and 24 hours post-infection and the bacteria enumerated by culture.

Significant numbers of *P*. *gingivalis* were able to be recovered from naive, M1 and M2 macrophages after 1 hour incubation at all BMRs tested (**Fig 3A**) However at 1000:1 BMR the numbers of bacteria recovered were significantly less than at both 10:1 and 100:1 (**Fig 3A**). As demonstrated in further experiments, the addition of high cell numbers of bacteria, such as 1000:1 BMR, induced apoptosis in all macrophage subtypes, which would affect functions such as phagocytosis (**Fig 3A**). In this assay the M1 and M2 phenotypes contained less viable bacteria than the naive subset, which differed from the pHrodo assay and the microscopy investigation. However, as both the pHrodo assay and the microscopy did not depend on the viability of the bacterial cells, differences were not unexpected and helped provide further insight into phagocytosis and survival of *P*. *gingivalis* in macrophage subsets. Interestingly, the inhibition of phagocytosis with cytochalasin B resulted in no *P*. *gingivalis* invasion of naive, M1 or M2 macrophages. The survival assay demonstrated that significant amounts of *P*. *gingivalis* were able to persist after 24 hours (**Fig 3B**). The results of the survival assay are expressed as a percentage of the cells that had invaded after 1 hour, and indicates that M1 macrophages were able to clear a significant fraction of the invaded bacteria after 24 hours. M2 macrophages however, were not significantly different from the naive macrophages, with both phenotypes containing similar amounts of viable *P*. *gingivalis* at all BMRs tested.

### M1 macrophages, but not naive or M2 macrophages, produce high levels of TNF-alpha, IL-12 and nitric oxide

**Fig 4:**
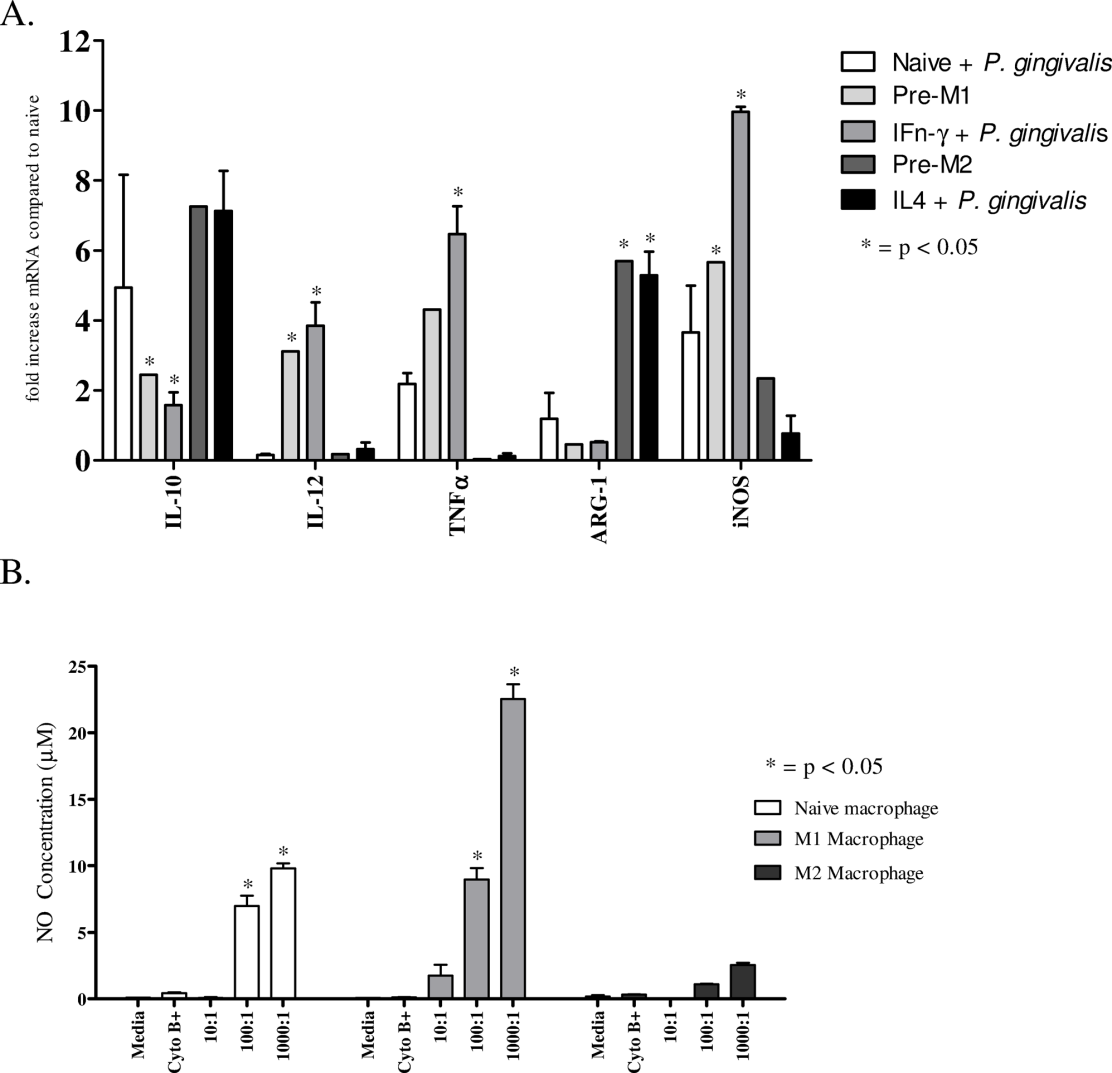
Cytokine mRNA and nitrite levels obtained from naive, M1 and M2 macrophages. Cytokine primed macrophages were incubated with *P*. *gingivalis* W50 (Bacteria to cell ratio 100:1) for 90 minutes at 37°C and supernatant was collected 24 hours post infection for nitric oxide (nitrite metabolite) and cells were lysed for mRNA extraction. **(A)** Cytokine concentration was determined using RT-PCR. **(B) N**itric oxide (NO) secretion by naive, M1 and M2 macrophages was measured indirectly by quantifying the level of nitrite (metabolite) in cell supernatants. Cytochalasin B was added as negative control as it stabilises microtubules and inhibit actin polymerisation, a process required for phagocytosis. Data are representative of independent experiments and triplicate biological samples are expressed as the mean ± standard deviation (n = 3) and were analysed using student's t-test. * indicates data that are significantly different (*p* < 0.05) from the data for non-stimulated group (media and macrophage alone group).

In order to characterise the cytokine profiles of naive, M1 and M2 macrophages in response to stimulation with *P*. *gingivalis*, RNA was extracted from macrophages incubated with or without IL-4 or IFN-γ and then infected with *P*. *gingivalis* (**Fig 4A**).

Incubation of naive macrophages with *P*. *gingivalis* resulted in the upregulation of moderate levels of IL-10, iNOS and TNF-α mRNA (**Fig 4A**). The addition of IFN-γ to the naive macrophages alone upregulated the synthesis of IL-12, iNOS and TNF-α, all markers of M1 macrophages, however this upregulation was significantly more pronounced after the addition of *P*. *gingivalis* to the primed macrophages. Priming of macrophages with IL-4 increased the synthesis of IL-10 and ARG-1, both markers of M2 macrophages; however the addition of *P*. *gingivalis* to IL-4 primed macrophages did not further increase the expression of these genes. ARG-1 and iNOS mRNA analyses were included in this study to confirm the generation of the correct macrophage phenotype.

The production of nitric oxide in M1 macrophages is a characteristic marker of classical activation and an indicator of the bactericidal capacity of the activated macrophage. The Greiss reaction is a common method of indirectly measuring nitric oxide production by quantifying the major metabolite of NO, nitrite. Naive macrophages in this assay were able to produce low levels of nitric oxide in response to *P*. *gingivalis* (**Fig 4B**). M1 macrophages produced significantly more nitric oxide than naive or M2 macrophages, and this correlated with the decreased persistence of *P*. *gingivalis* within the phagosome of M1 macrophages. Further, inhibition of phagocytosis by incubation with cytochalasin B abrogated nitric oxide production induced by *P*. *gingivalis*, indicating phagocytosis is critical for *P*. *gingivalis*-induced nitric oxide secretion. Urea assays were also performed on naive, M1 and M2 macrophages, however the generation of urea was solely dependent on the addition of IL-4. The addition of *P*. *gingivalis* did not further increase the concentration of urea present (data not shown).

### Infection of macrophage phenotypes with *P*. *gingivalis* induces significant levels of apoptosis

**Fig 5:**
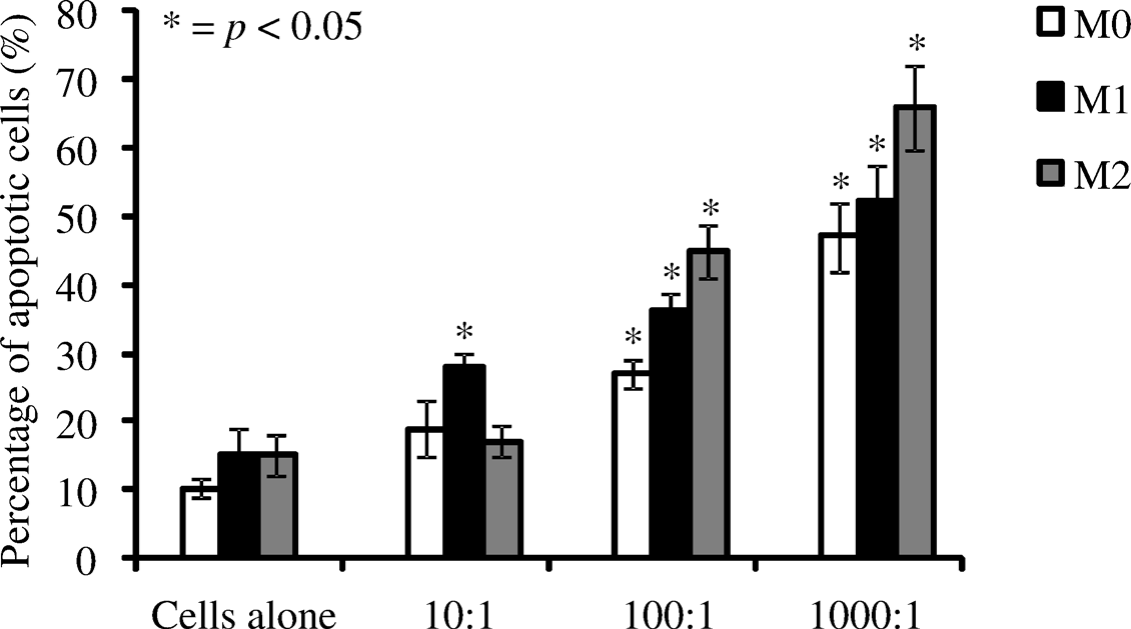
The effect of concentration of *P*. *gingivalis* on naive, M1 and M2 macrophage apoptosis. The percentage of apoptotic naive, M1 and M2 macrophages after incubation with *P*. *gingivalis* (bacteria to macrophage ratio– 10: 1, 100:1 and 1000:1) for 90 minutes, 37°C. The negative controls were naive, M1 and M2 macrophages alone. Apoptosis was detected using YO-PRO®-1 dyes and flow cytometric analysis. Data are representative of independent experiments and triplicate biological samples are expressed as the mean ± standard deviation (n = 3) and were analysed using student's t-test. * indicates data that are significantly different (*p* < 0.05) from the data for non-stimulated group (macrophage alone group).

A further possible consequence of bacterial invasion of, or phagocytosis by, macrophages is controlled apoptotic death of the macrophage. The ability of live *P*. *gingivalis* W50 to induce apoptosis of naive, M1 and M2 after 90 minutes incubation was investigated at several BMRs. *P*. *gingivalis* W50 induced dose-dependent apoptosis in macrophages (**Fig 5A**). A BMR of 10:1 induced very low levels of apoptosis in macrophages, with only M1 macrophages undergoing significant apoptotic death. However at higher doses of 1000:1, a very high percentage of all macrophage phenotypes were found to be apoptotic. Generally, *P*. *gingivalis* induced higher levels of apoptosis in M2 macrophages compared with the other subsets tested.

## Discussion

Macrophages play an essential role in the initiation and maintenance of inflammation [28]. The plasticity of macrophages, their ability to adapt to different cytokine stimuli and alter their functional properties, also makes macrophages essential in the control and resolution of inflammation [29–31]. We have previously demonstrated that CD86(+) macrophages producing nitric oxide (M1) are the primary phenotype generated in the gingival tissue during an *in vivo* infection with *P*. *gingivalis* [23], however to date no study had addressed the specific interactions of macrophage subsets with *P*. *gingivalis* cells. The objective of this study was to investigate the ability of naive, M1 and M2 macrophages to phagocytose *P*. *gingivalis* and the effects that phagocytosis has on both the macrophage and the bacterial cell.

*P*. *gingivalis* labelled with the pH sensitive dye pHrodo is an effective way to measure the phagocytosis of bacteria by macrophages without the complication of external adherent bacteria. Priming of macrophages with both IFN-γ and IL-4 increased both the percentage of pHrodo positive macrophages and the MFI of the positive macrophages, indicating an increase phagocytic ability. The increased phagocytosis by M1 and M2 macrophages observed by flow-cytometry in this study was confirmed by super-resolution microscopy, which also demonstrated the varied morphology of the different macrophage subsets. It has been shown previously that forcing macrophages to conform to an elongated, fibroblast-like shape induces the same macrophage phenotype as IL-4 or IL-13 treatment, suggesting that this morphology is associated with M2 macrophages performing their anti-inflammatory, tissue-repair function [32]. There were differences in the uptake of *P*. *gingivalis* depending on the method used to measure phagocytosis. The pHrodo assays indicated that M1 macrophages phagocytosed *P*. *gingivalis* at a significantly higher level compared to naive and M2 macrophages, both at 20:1 BMR and reflected in the MFI at higher BMRs. However the results from the super-resolution microscopy suggested that M2 macrophages had higher levels of internalised bacteria than both M1 and naive macrophages. It is possible that the dependence of pHrodo on acidification of the phagolysosome influenced the result in M2 macrophages. This would be particularly evident if *P*. *gingivalis* was preferentially invading M2 macrophages and persisting in the cytoplasm rather than a phagosome, an effect that has been previously demonstrated in gingival epithelial cells [33]. Persistence outside the phagosome would render the bacteria undetectable by pHrodo fluorescence, however the cells would still appear when visualised with a conventional fluorophore such as AF488 and would still be viable for culture in the invasion assay.

To investigate the fate of *P*. *gingivalis* cells after phagocytosis by IFN-γ and IL-4 primed macrophages, we performed both a macrophage invasion assay and a survival assay. Naive, M1 or M2 were exposed to *P*. *gingivalis* and then the macrophages were lysed and the number of viable *P*. *gingivalis* enumerated after 1 hour (invasion) or 24 hours (survival). Previously it has been reported that *P*. *gingivalis* can survive within macrophage phagosomes [34, 35]. We found 10–30% of the initial invaded bacteria were present in naive and M2 macrophages after 24 hours. However M1 macrophages had cleared significantly more bacteria than M2 and naive macrophages, which correlated with the increased nitric oxide production observed. As *P*. *gingivalis* W50 lacks functional FimA fimbriae, which are the cellular structure hypothesised to be responsible for both increased phagocytosis of *P*. *gingivalis* and intracellular survival within the macrophage [34, 35], it is unclear whether fimbriated strains of *P*. *gingivalis* would be able to survive to a higher degree. We have recently shown that the presence of fimbriae on *P*. *gingivalis* strains increases phagocytosis by several immune cells, and that the atypical, long fimbriae of *P*. *gingivalis* 33277 increased the MFI of the phagocytic immune cells, indicating a more robust phagocytic response [36]. Interestingly, *P*. *gingivalis* W50 and other W50-like strains have been more closely associated with disease and as such the study of the immune response to this strain is important [37, 38].

Even though IFN-γ primed (M1) macrophages displayed the highest phagocytosis in the pHrodo assay, they contained about the same level of viable bacteria as the naive group following a 1 hour incubation. Differences in the acidity of the phagosome or the intracellular location of *P*. *gingivalis* may help explain this result. However, as the M1 cells are able to produce significant amounts of nitric oxide, as demonstrated in this study, they were able to kill significantly more *P*. *gingivalis* by 24 hours. In contrast, IL-4 primed (M2) macrophages while initially containing fewer internalised *P*. *gingivalis* than IFN-γ primed cells at 1 hour were unable to completely clear the infection by 24 hours. This demonstrates the decreased anti-microbial functions of M2 macrophages relative to the IFN-γ primed M1 macrophages.

Phagocytosis of bacterial pathogens can also have a deleterious effect on the macrophage, inducing apoptosis [39, 40]. We used a combination of Yo-Pro and Propidium iodide dyes to determine early stages of apoptosis and cell death in macrophage subsets after infection. *P*. *gingivalis* induced apoptosis in a dose dependent manner, with roughly a third of macrophages being apoptotic at a BMR of 100:1 and up to two thirds apoptotic at a BMR of 1000:1. *P*. *gingivalis* contains many virulence factors to aid in the acquisition of nutrients in a biofilm accreted to the tooth root [41], and is therefore aided by the death and lysis of host cells with a subsequent release of host nutrients. The induction of apoptosis in host cells could further contribute to the immune dysregulation observed during chronic periodontitis [41, 42].

Inflammatory cytokine TNF−α and IL-12 mRNA were produced by M1 macrophages in response to *P*. *gingivalis*, whereas unprimed and M2 macrophages only produced low levels of inflammatory cytokine mRNA. Other investigations have found that *P*. *gingivalis* cells, or cell components, induce the production of significant amounts of inflammatory cytokines in naive macrophages [43–45]. However few investigations have used macrophages primed with cytokines. Considering the level of cytokines present in the gingival tissue during active chronic periodontitis it is conceivable that very few macrophages would encounter TLR ligation in the absence of pro-inflammatory cytokine stimulation. Therefore the experiments presented here better reflect the contribution of macrophages to inflammation during chronic periodontitis. Several pro-inflammatory cytokines are generally elevated during chronic periodontitis, TNF−α being amongst the most prominent [46, 47]. The results here suggest that macrophages (M1 in particular), may contribute substantially to the TNF-α produced during disease. This is consistent with our previous findings of M1 macrophages being the most prevalent macrophage phenotype during oral infection of mice with *P*. *gingivalis* [23]. The data presented in this study demonstrate that priming macrophages with IFN-α or IL-4 increased their phagocytic capacity. However, only priming with IFN-γ induced the production of significant amounts of nitric oxide, which correlated with an enhanced bactericidal capability. Further, M1 macrophages responded to infection with *P*. *gingivalis* with the production of TNF-α and IL-12, both cytokines important in initiating and regulating inflammation. It is possible then that IFN-γ produced during infection with *P*. *gingivalis* shifts the macrophage differentiation to a predominant M1 phenotype. This is consistent with our previous results showing the effect of isolated *P*. *gingivalis* LPS on macrophage phenotypes and the increased prevalence of M1 macrophages in gingival tissue of mice infected with *P*. *gingivalis* [19, 23]. This was also recently confirmed in a ligature model of periodontal disease which demonstrated a greater increase in M1 macrophage markers over M2 markers in the periodontal tissues of mice [48]. We propose based on all the data that this predominantly M1 phenotype should be able to phagocytose and kill *P*. *gingivalis* when at low BMRs distal to the site of infection while producing localised inflammation through the production of inflammatory cytokines. However as the BMR increases closer to the plaque biofilm, M1 macrophages would be exposed to high levels of bacterial products, inducing apoptosis and compromising their antimicrobial function. M2 macrophages are less dominant in gingival tissue of mice infected with *P*. *gingivalis*. This may not be unexpected given the phenotypic plasticity of macrophages and the inflammatory environment in chronic periodontitis. However given that CD163, an M2 macrophage marker, may also be a biomarker of healthy gingival tissue [49] the role of M2 macrophages in the progression of chronic periodontitis remains unclear.

## Materials and Methods

### Bacterial strains and growth conditions

*P*. *gingivalis* strain W50 (ATCC 53978) was obtained from the culture collection of the Oral Health Cooperative Research Centre at the Melbourne Dental School. *P*. *gingivalis* W50 was grown and harvested as described previously [23, 50–52]. Growth conditions of batch cultures were monitored at 650 nm using a spectrophotometer (model 295E, Perkin-Elmer, Germany). Cells were harvested during late exponential growth by centrifugation (7000 g, 20 minutes at 4°C).

### Labelling of *P*. *gingivalis* with Alexa-Flour 488 (AF 488) and pHrodo-Red

*P*. *gingivalis* W50 grown to late exponential growth was washed once (8000 g, 20 minutes) in 0.1 M Phosphate-buffered saline (PBS—0.01 M Na_2_HPO_4_, 1.5 mM KH_2_PO_4_, 0.15 M NaCl–pH 7.2) and incubated with 100 μg/mL Alexa-Fluor 488 (AF 488–FITC) (Life Technologies Pty Ltd, NSW, Australia) for 1 hour, 37°C shaking incubator in the dark. Suspensions were then washed (3 x) in PBS and the number of AFF488 labelled *P*. *gingivalis* W50 (number/mL) were determined by flow cytometry using a Cell Lab Quanta SC flow cytometer (Beckman Coulter Pty Ltd, NSW, Australia) and a LIVE/DEAD BacLight™ Bacterial Viability Kit, according to the manufactures instructions (Life Technologies Pty Ltd, NSW, Australia). For pHrodo-Red^TM^ labeling (Life Technologies Pty Ltd, NSW, Australia), *P*. *gingivalis* W50 grown to late exponential growth was washed once (8000 g, 20 minutes) were resuspended in 100 mM sodium bicarbonate, pH 8.5 at 3 x 10^9^ cells/mL. The bacteria were incubated with pHrodo at a concentration of 0.5 mM for 1 hour at room temperature (RT) in the dark, with gentle mixing. Labelled bacteria were then washed (3 x) in PBS and resuspended at 3 x 10^9^ cells/mL in PBS before being snap frozen in liquid nitrogen and stored at -80°C.

### Cell Lines and Media

All cell culture reagents were obtained from Sigma-Aldrich Pty. Ltd. (NSW, Australia) unless specified. RAW 264.7 cells, a murine macrophage cell line were maintained as frozen stocks and cultured in complete Dulbecco’s Modified Eagle Medium (DMEM) supplemented with 10% v/v heat-inactivated FBS (30 minutes, 56°C), 2 mM L-glutamine, 2 mM sodium pyruvate, 0.1 mM 2-ME, 30 g/mL gentamicin and 100 IU/mL penicillin (1 hour, 37°C). RAW 264.7 cells are designated as naive in this experiment.

### Generation M1 and M2 macrophages

To prime macrophages, RAW 264.7 cells were incubated overnight in complete DMEM supplemented with either 20 U/mL of IFN-γ (ABD Serotec, Oxford, United Kingdom) or 150 U/mL of IL-4 (ABD Serotec, Oxford, United Kingdom) to generate pre-M1 and pre-M2 macrophages, respectively (as previously described) [19]. Naive macrophages treated with IFN-γor IL-4 but not any TLR stimulant are defined as pre-M1 or pre-M2 macrophages, respectively.

### Phagocytosis Assay

pHrodo-Red^TM^ (Life Technologies Pty Ltd, NSW, Australia) is a novel pH sensitive fluorochrome that has been used to examine phagocytosis. RAW 264.7 cells (naive, pre- M1 and pre-M2) were used to examine phagocytosis of *P*. *gingivalis* W50. Adherent macrophages were removed using 0.25% trypsin-EDTA and resuspended in antibiotic and serum free DMEM at 1.5 x 10^6^ cells/mL. pHrodo-Red labelled *P*. *gingivalis* were added in 3 different bacteria to cell ratios (20:1, 80:1, 160:1) in triplicate (n = 3) and incubated for either 15 minutes or 60 minutes at 37°C, 5% v/v CO_2_. After incubation the cells were placed on ice to stop phagocytosis then washed twice and resuspended in sterile ice cold PBS for analysis by flow cytometry on the FC500 (Beckman Coulter Australia, Gladesville, NSW, Australia). A typical forward and side-scatter gate was set to exclude dead cells and aggregates; a total of 3 x 10^4^ events in the gate were collected and phagocytosis was identified as pHrodo-Red positive cells (pHrodo-Red fluorescence was measured using 575 nm filter; FL2). Macrophages and bacteria incubated on ice rather than at 37°C were used as negative controls.

### Super-resolution imaging

To visualise the phagocytosis of *P*. *gingivalis* W50 by naive, pre-M1 and pre-M2 macrophages, 1 x 10^5^ RAW 264.7 cells/per slide were grown and primed in chambered coverglass slides (Thermo Fisher Scientific, Scoresby, VIC, Australia) as described above. The cells were washed with PBS (2 x) and incubated (1hour, 37°C) in the dark with AF488 labelled *P*. *gingivalis* W50 (bacteria to macrophages ratio of 60:1) in serum and antibiotic free DMEM. Macrophages were then washed with PBS (1x) and fixed with 4% paraformaldehyde for 5 minutes at room temperature (RT). The macrophages were washed with PBS (2 x) and then permeabilised with 0.1% Triton X-100 (Sigma-Aldrich Pty. Ltd., NSW, Australia) in PBS at RT. Following washing with PBS (2 x), the macrophages were blocked with 1% BSA in PBS at RT for 5 minutes. The actin filaments were stained with Phalloidin-TRITC (Sigma-Aldrich Pty. Ltd., NSW, Australia) at 5 μg/mL in 1% BSA/PBS for 40 minutes, 37°C, and nuclei were stained with DAPI as per manufacturer’s instructions (Life Technologies Pty Ltd, NSW, Australia). The macrophages were stored in SlowFade Diamond Antifade Mountant (Life Technologies Pty Ltd, NSW, Australia) before imaging on the Deltavision OMX Structured Illumination Microscope V4 Blaze (Applied Precision, WA, USA). Images were produced using Fiji imaging processing package [53].

### Invasion Assay

RAW 264.7 macrophages (1 x 10^5^ naive, primed M1 and M2 macrophages) were seeded into 96-well plates. Each treatment was seeded in triplicate biological samples (n = 3) and one of the groups was incubated with 5 μg/mL cytochalasin B (Sigma-Aldrich Pty. Ltd., NSW, Australia) for 60 minutes at 37°C. Cytochalasin B was added as a negative control as it stabilises microtubules and inhibit actin polymerisation, a process required for invasion [54]. The macrophages were washed with serum and antibiotic free DMEM (2 x) and then incubated with *P*. *gingivalis* W50 in serum and antibiotic free DMEM (bacteria to macrophage ratios– 10: 1, 100:1 and 1000:1 for 90 minutes, 37°C). The supernatant were discarded and macrophages washed with DMEM (2 x). External adherent bacteria and those that were not removed by washing were killed by incubation for 1 hour with constant gentle shaking in DMEM supplemented with 300 μg/mL gentamicin and 200 μg/mL metronidazole. These concentrations of antibiotics were sufficient to kill 10^9^ bacterial cells per mL in 1 hour [33]. After incubation with antibiotics, the macrophages were then washed with serum and antibiotic DMEM (2 x). The naive, M1 and M2 macrophages with internalised bacteria were then lysed with cell lysis solution (1% Triton X-100/PBS) (Sigma-Aldrich Pty. Ltd., NSW, Australia). All wells were monitored under the microscope to ensure that all cells were completely lysed (not sticking on the bottom of the well) before 100 μL of the lysed cells containing *P*. *gingivalis* were transferred to 900 μL brain heart infusion (BHI) medium or H_2_0 respectively. Another identical plate was set up where upon removal of antibiotics, the macrophages were washed in DMEM containing antibiotics and 10% FBS (2 x) and incubated for a further 24 hours before the lysing step. An aliquot of the supernatant from the post 24 hours samples were also collected to determine cytokines and the nitric oxide concentration produced by naive, M1 and M2 macrophages using Bioplex and Greiss reagent kit, respectively. Internalised *P*. *gingivalis* were plated at various dilutions using the drop plate method on HBA or LB agar respectively and colonies forming units (cfu) enumerated after incubation (under anaerobic incubation at 37°C for 6 days and under aerobic condition at 37°C for 1 day).

### Determination of cytokines induced in macrophages 4 hours-post infection with *P*. *gingivalis* using RT-PCR

RAW264 macrophages were incubated overnight in the presence or absence or 50ng/mL IFN-γ (M1) or 100ng/mL IL-4 (M2). *P*. *gingivalis* W50 was added to the macrophages (100:1 BMR) and incubated for 4 hours at 37°C. Total RNA was extracted using the RNeasy plus RNA extraction kit (Qiagen). Following stabilisation in RNAProtect (Qiagen), buffer RLT (350 μL) was added and the solution homogenised by centrigugation in 10000 x g in a Qiashredder for 2 minutes. Ethanol (70% w/v, 350 μL) was then added and the entire 700 μL was added the RNeasy spin column and centrifuged at 16000 x g for 1 minute. The membrane was washed with 700 μL of buffer RW1 followed by two washes with 500 μL of buffer RPE. A final centrifugation at 16000 x g for 1 minute was performed to dry the membrane. RNase free water (30 μL) was added to the membrane and the RNA was eluted by centrifugation at 16000 x g for 1 minute. RNA was quantified by absorbance (260 nm/280 nm) using a Nanodrop spectrophotometer (Thermo Scientific)

Extracted RNA was immediately DNase treated using the Turbo DNA free kit (Ambion). Briefly, 5 μg of RNA was combined with 2 μL of 10X DNase buffer, 1 μL of DNase and sufficient water to make up a 20 μL reaction volume. The reaction was incubated for 20 minutes at 37°C, after which a further 1 μL of DNase was added and the reaction incubated for another 20 minutes at 37°C. Following this second incubation, 2 μL of DNase inactivationreagent was added. After a 5 minute incubation at RT with occasional mixing, the inactivation reagent was pelleted by centrifugation at 10000 x g for 1 minute and the supernatant collected.

Reverse transcription was performed using the iScript reverse transcription supermix (Biorad). Briefly, 1 μg (4 μL) of the DNase treated RNA extraction was combined with 4 μL of iScript master mix and 12 μL of RNase free water. The RT reaction was performed with a 5 minute, 25°C priming step, a 30 minute, 42°C extension step followed by a 5 minute, 85°C inactivation step. A no-RT reaction was also setup up using 1 μg (4 μL) of the DNase treated RNA extraction combined with 16 μL of RNase free water.

Real Time PCR was performed using the iTaq Universal Sybr Green Supermix (Biorad). Template cDNA (25 ng, 1 μL) was combined with 0.8 μL of Forward primer (200 nM final), 0.8 μL of Reverse Primer (200 nM final) and 10 μL of Sybr Green Supermix. Thermal cycling was performed using 40 cycles of 95°C for 15 seconds, 52°C for 15 seconds and 72°C for 15 seconds. A positive control of genomic DNA and a no-RT control was included in each cycling run. Cycling was performed on a Rotor Gene RG-3000A Thermal Cycler running Rotor-Gene V6.1 software (Corbett Research). Analysis of the PCR was performed using the LinRegPCR software version 2015.3. Comparative CT analysis was performed according to the method of Schmittgen and Livak [55].

### Measurement of Nitrite in cell supernatants

Nitrite concentration was determined using the Greiss reagent kit (Life Technologies Pty. Ltd., NSW, Australia) according to the manufacturer’s instructions. Briefly, 150 μL of naive, M1 and M2 macrophage culture supernatant from *P*. *gingivalis* stimulated cells (1 hour stimulation, bacteria to macrophage ratio 10:1, 100:1 and 1000:1) in triplicate which were collected after 24 h incubation, was combined with 130 μL of deionised water, 10 μL of N-(1-naphthyl)ethylenediamine dihydrochloric (1 mg/mL) and 10 μL of Sulfanilic acid (1.0 mM). A standard curve was generated using 2-fold serial dilutions of a 100 μM nitrite standard solution (100 μM to 1.56 μM). The reaction was allowed to proceed (30 minutes, RT) and the absorbance was measured at 550 nm using Victor3 1420 multilabel counter (Perkin Elmner, Massachusetts, USA). Negative control was naive cell alone.

### Apoptosis Assay

RAW 264.7 macrophages (1 x 10^5^ naive, primed M1 and M2 macrophages) were seeded at 2 x 10^6^ cells/200μL in FACS tubes and incubated with *P*. *gingivalis* (bacteria to macrophage ratio– 10: 1, 100:1 and 1000:1 for 90 min, 37°C). The negative controls were naive, M1 and M2 macrophages alone. The macrophages were washed in fluorescence activated cell sorting buffer (FACS buffer) (24% w/v BSA, 0.1% w/v sodium-Azide, 20mM EDTA in Dulbecco’s PBS) (2 x). Naive, M1 and M2 cells then incubated with 1/1000 of Yo-Pro and PI stock solution in Vybrant® Apoptosis Assay Kit, YO-PRO®-1/Propidium Iodide kit as per manufacturer’s instructions (Life Technologies Pty Ltd, NSW, Australia) for 20 minutes on ice. The stained cells were analysed straight after incubation using flow cytometry on the FC500 (Beckman Coulter Australia, Gladesville, NSW, Australia). A total of 3 x 10^4^ events in the gate were collected and apoptotic cells were identified as Yo-Pro positive cells while necrotic cells were identified as PI positive cells according to manufacturer’s settings.

### Statistical analysis

All data were statistically analysed using an ANOVA with a Bonferroni post-test and *p* < 0.05 was considered significant. All statistical analyses were performed using Graphpad Prism v5.0.
